# Dermoscopic Features of a Black Hairy Tongue in 2 Japanese Patients

**DOI:** 10.1155/2010/145878

**Published:** 2010-07-26

**Authors:** Ken Kobayashi, Yasuhiko Takei, Mizuki Sawada, Sumiko Ishizaki, Haruo Ito, Masaru Tanaka

**Affiliations:** ^1^Department of Dermatology, Tokyo Women's Medical University Medical Center East, 2-1-10, Nishi-Ogu, Arakawa-ku, Tokyo 116-8567, Japan; ^2^Department of Otolaryngology, Kyorin University School of of Medicine, Tokyo 181-8611, Japan

## Abstract

Dermoscopic features of a black hairy tongue have never been reported. Dermoscopy might be useful in speculating pathologic features of oral lesions. The objective was to identify additional dermoscopic criteria. Two Japanese patients who were clinically given a diagnosis of “black hairy tongue” were evaluated for dermoscopic features. We have shown characteristic dermoscopic features of brownish hair-like elongation of filiform papillae with whitish lingual papillae. Dermoscopic examination seemed useful as an adjunct to the diagnosis of this benign disorder of the tongue, demonstrating exact changes in shape and color of filiform papillae. It might also be helpful in more objective observation of the therapeutic efficacy.

Black hairy tongue is a benign disorder characterized by hypertrophy of the filiform papillae of the tongue and is usually asymptomatic. A common clinical feature of a black hairy tongue is brownish-black discoloration caused by variety of precipitating factors, such as chronic smoking, poor oral hygiene, tooth loss, chronic or extensive use of antibiotics, and radiation therapy to the head and neck. Although the etiology of black hairy tongue is not well understood, secondary infection of *candida albicans *and/or *bacillus subtilis varietas niger * can frequently be involved. 

We report two typical cases of black hairy tongue with dermoscopic findings. 

 Two Japanese patients, who were diagnosed as having a black hairy tongue, were evaluated for dermoscopic features using nonpolarized, contact-type dermoscopic instrument with echo gel, Derma9500 (DMI Inc., Yokohama, Japan) in combination with PowerShot 630A (Canon, Tokyo, Japan). KOH-prepared direct microscopic examination in two cases and bacterial cultivation in a case were also performed.

**Case 1**

An 82-year-old Japanese man of complained of dysgeusia with black discoloration on the tongue surface (Figure 1(a)). Physical examination revealed yellowish brown to brownish black discoloration on the dorsum of the tongue. KOH-prepared direct microscopy demonstrated no fungal elements. Dermoscopic examination demonstrated brownish hair-like elongation of filiform papillae with whitish lingual papillae (Figure 1(b)). The patient was treated with oral miconazole gel, and most of the oral lesion abated promptly after the treatment.

**Case 2**

A 77-year-old Japanese woman complained of hyperesthesia of the tongue with brown colored discoloration occurring about three months after a treatment of oral candidiasis. Physical examination revealed yellowish brown to brownish black discoloration on the dorsum of the tongue (Figure 2(a)). KOH-prepared direct microscopy demonstrated fungal elements. Dermoscopic examination demonstrated numerous brownish hair-like elongation of filiform papillae covering over whitish lingual papillae (Figure 2(b)). *Candida albicans* was isolated and identified by cultivation on ATG ager. The patient was treated with oral itraconazole and most of black discoloration of the tongue subsided promptly after the treatment.

Abnormal hair-like elongation of filiform papillae of the middorsal tongue is an acquired condition seen most frequently in chronic smokers [1]. The characteristic appearance is usually restricted to the dorsum of the tongue, immediately anterior to the circumvallate papillae. The “hair-like” appearance results from elongation of the filiform papillae. The color of the papillae ranges from yellowish brown to brownish black. A scanning electron microscopy studies have shown that the lengthening of the filiform papillae is due to accumulated keratinized layer [2]. Generally, ultrastructural architecture of black hairy tongue epithelium reveals significant elongation of filiform papillae filled with “hair” keratins. These findings are highly different from normal filiform papillae. 

As dermoscopic observation is not vertical but horizontal, dermoscopic features efficiently demonstrated yellow-brownish to black hair-like elongation of filiform papillae with whitish lingual papillae, clearly. 

Although dermoscopic features of a tongue lesions were seldom reported, dermoscopy might be useful in speculating pathologic features in the tongue, such as this lesion. In this report, we have shown characteristic dermoscopic features of black hairy tongue. Dermoscopic examination seemed useful as an adjunct to a diagnosis of this benign disorder of the tongue, demonstrating exact changes in shape and color of filiform papillae. It might also be helpful in more objective observation of the therapeutic efficacy. Evaluations of tongue using dermoscopy would deepen understanding of various other dermoscopic features in the lingual papillae, such as coating of the tongue, median rhomboid glossitis or thrush and might be useful for the differential diagnoses in the clinical practice and investigations.

## Figures and Tables

**Figure 1:**
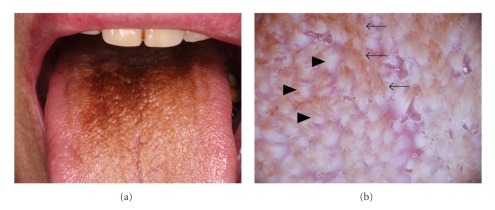
(a) Black hairy tongue on the dorsum of the tongue (Case 1). (b) Brownish hair-like elongation of filiform papillae (→) and whitish lingual papillae (▲) (Case 1).

**Figure 2:**
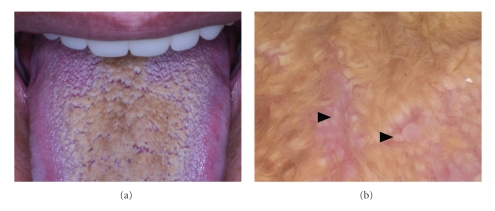
(a) Black hairy tongue on the dorsum of the tongue (Case 2). (b) Numerous brownish hair-like elongation of filiform papillae covering over whitish lingual papillae (▲) (Case 2).
